# Prognostic value of central blood pressure on the outcomes of embolic stroke of undetermined source

**DOI:** 10.1038/s41598-023-36151-y

**Published:** 2023-06-12

**Authors:** Minho Han, JoonNyung Heo, Il Hyung Lee, Joon Ho Kim, Hyungwoo Lee, Jae Wook Jung, In Hwan Lim, Soon-Ho Hong, Young Dae Kim, Hyo Suk Nam

**Affiliations:** 1 Department of Neurology, Yonsei University College of Medicine, 50-1 Yonsei-Ro, Seodaemoon-Gu, Seoul, 03722 South Korea; 2 Integrative Research Center for Cerebrovascular and Cardiovascular Diseases, Yonsei University College of Medicine, 50-1 Yonsei-Ro, Seodaemoon-Gu, Seoul, 03722 South Korea

**Keywords:** Diseases, Neurology, Risk factors

## Abstract

We investigated the prognostic impact of central blood pressure (BP) on outcomes in patients with embolic stroke of undetermined source (ESUS). The prognostic value of central BP according to ESUS subtype was also evaluated. We recruited patients with ESUS and data on their central BP parameters (central systolic BP [SBP], central diastolic BP [DBP], central pulse pressure [PP], augmentation pressure [AP], and augmentation index [AIx]) during admission. ESUS subtype classification was arteriogenic embolism, minor cardioembolism, two or more causes, and no cause. Major adverse cardiovascular event (MACE) was defined as recurrent stroke, acute coronary syndrome, hospitalization for heart failure, or death. Over a median of 45.8 months, 746 patients with ESUS were enrolled and followed up. Patients had a mean age of 62.8 years, and 62.2% were male. Multivariable Cox regression analysis showed that central SBP and PP were associated with MACE. All-cause mortality was independently associated with AIx. In patients with no cause ESUS, central SBP and PP, AP, and AIx were independently associated with MACE. AP and AIx were independently associated with all-cause mortality (all *p* < 0.05). We demonstrated that central BP can predict poor long-term prognosis in patients with ESUS, especially those with the no cause ESUS subtype.

## Introduction

Embolic stroke of undetermined source (ESUS) is a non-lacunar ischemic stroke without apparent arterial stenosis or major cardioembolic sources^1^. Of all patients with ischemic stroke, 9–25% are classified as ESUS^2^. Recurrent stroke and mortality rates of patients with ESUS are high at 3.9% and 4.5% per year, respectively^2,3^. Previous large randomized clinical trials found that treatment with direct oral anticoagulants of patients with ESUS was ineffective^4,5^. One reason might be the heterogeneity of ESUS^6^. Thus, a method of determining the prognosis according to ESUS subtype is needed. In addition, novel predictive markers for ESUS prognosis are warranted.

Prognosis of ESUS may be related to vascular risk factors such as blood pressure (BP). Central BP is the pressure in the aorta or the carotid artery. Central BP is more accurate and useful than peripheral BP because central BP may better represent the imposed load on the cerebral and coronary arteries. Furthermore, it shows a stronger relationship with vascular damage and the occurrence of cardiovascular diseases and mortality^7,8^. The Strong Heart Study supports this premise by demonstrating that central BP is a better predictor of future cardiovascular events than brachial pressure^9^. Central BP is also considered a clinical marker of silent cerebrovascular disease in an apparent general population^10,11^. Further studies have also shown that central BP is linked to poor short-term outcomes in patients with ischemic stroke^12,13^. However, the prognostic value of central BP for long-term outcomes in stroke, especially in patients with ESUS, has not been established.

In this regard, we hypothesized that hemodynamic parameters of central BP may predict poor long-term prognosis in patients with ESUS. We also investigated whether the association between central BP and long-term prognosis differed across ESUS subtypes.

## Methods

### Study population

From January 2012 to December 2018, patients with ESUS who were consecutively enrolled in the prospective registry were included. The definition of ESUS was based on the criteria proposed by the Cryptogenic Stroke/ESUS International Working Group^1^. Namely, patients with ESUS were defined as those who had non-lacunar infarctions, no cerebral artery stenosis, no major-risk cardioembolic sources (mechanical cardiac valve, mitral stenosis with atrial fibrillation, atrial fibrillation, left artrial/artrial appendage thrombus, sick sinus syndrome, recent myocardial infarction [< 4 weeks], left ventricular segment, atrial myxoma, infective endocarditis, atrial flutter, lone atrial fibrillation, bioprosthetic cardiac valve, mitral stenosis without atrial fibrillation, and nonbacterial thrombotic endocarditis), and no other uncommon causes (reversible cerebral artery vasoconstriction syndrome, vasculopathy, and cancer). During hospitalization, the patients underwent computed tomography, brain magnetic resonance imaging, and/or cerebral angiography. For standard evaluation, chest radiography, 12-lead electrocardiography, and blood tests, including lipid profiling, were performed. Central BP measurement and transesophageal echocardiography (TEE) were also performed as standard evaluations, except as otherwise restricted by applicable exclusion criteria^14^.

We classified patients with ESUS into (1) arteriogenic embolism, (2) minor cardioembolism, (3) two or more causes (arteriogenic embolism + minor cardioembolism), or (4) no cause. Arteriogenic embolism included complex aortic plaque (≥ 4 mm) and non-stenotic (< 50%) relevant artery plaque. Minor cardioembolism included mitral valve prolapse, mitral annulus calcification, left atrial turbulence (smoke), atrial septal aneurysm, patent foramen ovale, congestive heart failure, hypokinetic left ventricular segment, and myocardial infarction (> 4 weeks but < 6 months)^15^.

We collected information on baseline characteristics (age, sex, and National Institutes of Health Stroke Scale [NIHSS] score at admission), infarct location, risk factors, lipid profile, and secondary prevention. The presence of patent foramen ovale may affect stroke prognosis^16^ and was therefore included in the demographic characteristics. To obtain parameters for central BP, we performed pulse wave analysis of the radial artery using a commercially available applanation tonometer (SphygmoCor, Pulse Wave Analysis System, AtCor Medical, Sydney, Australia)^17^. Central BP parameters included central systolic BP (SBP), central diastolic BP (DBP), central pulse pressure (PP), augmentation pressure (AP), and augmentation index (AIx). AIx normalized to a heart rate of 75 beats per minute was used for analysis.

### Follow-up and outcomes

Patient follow-up was conducted at 3 months after discharge and annually via an outpatient clinic or structured telephone interview. The occurrence of a major adverse cardiovascular event (MACE) was the primary outcome and was defined as any occurrence of recurrent stroke, acute coronary syndrome, hospitalization for heart failure, or death. The secondary outcomes were recurrent stroke and all-cause mortality. Recurrent stroke was determined as a new neurologic deficit with relevant lesions on brain imaging 7 days after an index stroke or discharge. In addition, the date and cause of death were collected from the Korean National Statistical Office, which were determined based on the identification numbers of the death certificates. The censoring date was December 31, 2019. If the last visit occurred before that date, the last visit date was replaced with the censoring date^14^.

### Statistical analysis

Statistical analysis was performed using SPSS (version 26, IBM, Chicago, IL, USA) and R version 4.3.0 (http://www.r-project.org). Significant intergroup differences were analyzed by one-way ANOVA or the Kruskal–Wallis test for continuous variables and chi-square test or Fisher’s exact test for categorical variables. Data were presented as mean ± standard deviation or median (interquartile range) for continuous variables and number (%) for categorical variables. The cutoff values for central SBP, DBP, and PP were set at ≥ 130 mmHg, ≥ 90 mmHg, and > 50 mmHg, respectively, based on previous studies^18,19^. Because no cutoff values for AP and AIx have been previously reported, the cutoff values for AP and AIx were set at > 13 mmHg and > 25%, respectively, based on the median value. Survival curves were plotted using the Kaplan–Meier method and compared using the log-rank test. Multivariable Cox proportional hazard regression analysis was performed after adjusting for baseline characteristics (age, sex, and NIHSS score), ESUS subtype, and variables with *p* < 0.05 in univariable analysis. Each multivariable Cox regression analysis was also conducted according to the ESUS subtype. Restricted cubic spline curves were plotted to visualize the impact of central BP on prognosis. Two-tailed *p* values < 0.05 were considered statistically significant.

### Ethical approval

The Institutional Review Board of Severance Hospital of the Yonsei University Health System approved this study and waived the need for informed consent because of the retrospective design and observational nature of the study (Approval Number: 4-2021-1724). The study was conducted ethically in accordance with the World Medical Association Declaration of Helsinki.

## Results

### Demographic characteristics

**Figure 1:**
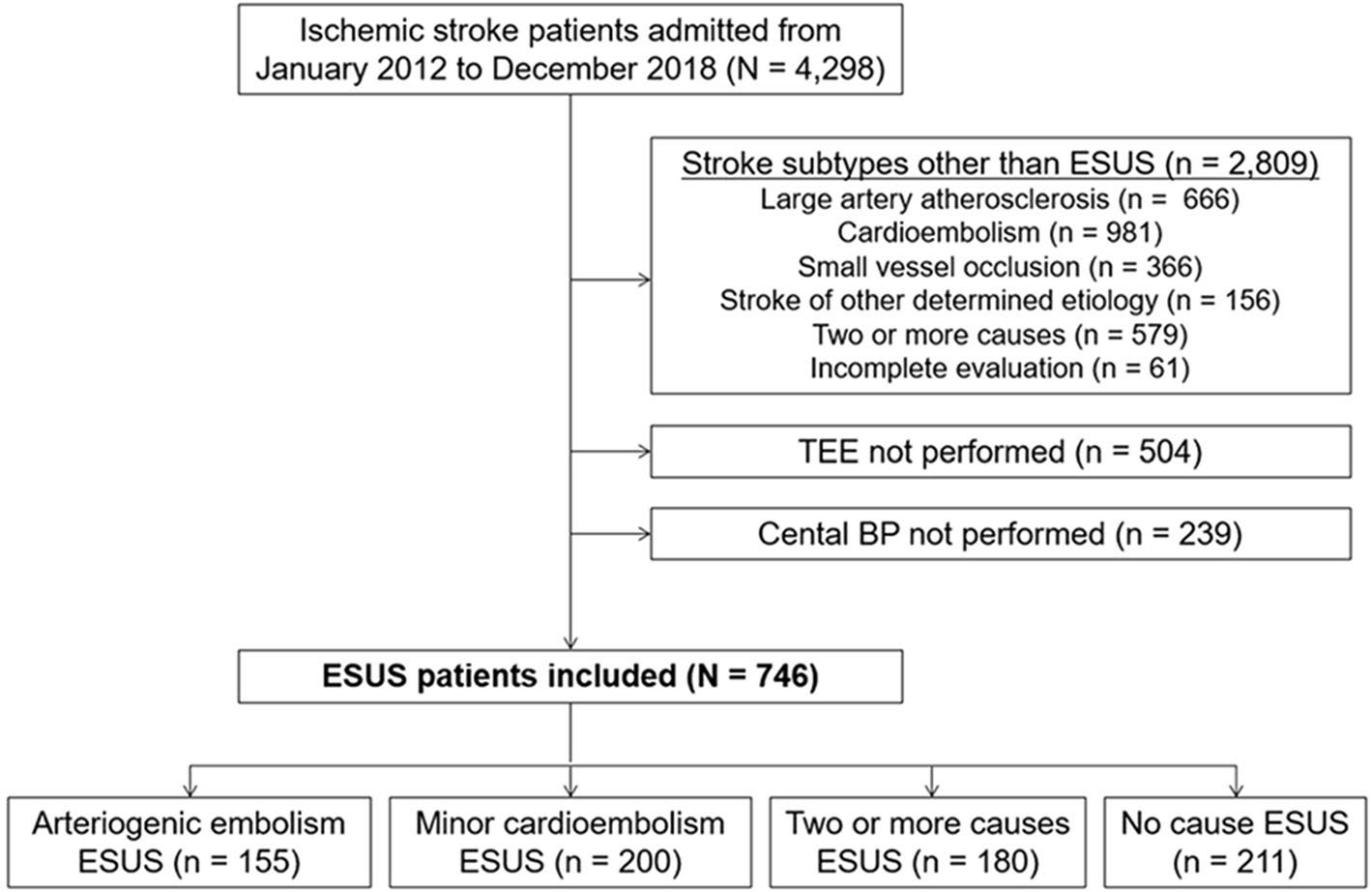
Patient flow chart. *BP* blood pressure, *ESUS* embolic stroke of undetermined source, *TEE* transesophageal echocardiography.

A total of 4,298 patients were registered during study period, we excluded those with stroke subtypes other than ESUS, including large artery atherosclerosis (n = 666), cardioembolism (n = 981), small vessel occlusion (n = 366), stroke of other etiology (n = 156), stroke with two or more causes (n = 579), and incomplete stroke evaluation (n = 61). Additionally, patients without TEE (n = 504) and central BP (n = 239) data were excluded. After exclusion, a final total of 746 patients with ESUS were included in this study, of which 155 (20.8%) had arteriogenic embolism, 200 (26.8%) had minor cardioembolism, 180 (24.1%) had two or more causes, and 211 (28.3%) had no cause (Fig. 1).

The patients had a mean age of 62.8 ± 13.1 years and a median NIHSS score of 2.0 (interquartile range, 1.0–4.0), and 464 (62.2%) were male. In terms of ESUS subtype, patients with arteriogenic embolism were the oldest, had the highest hypertension and diabetes rates, and had the highest central PP. Patients with two or more causes had the highest coronary artery disease rates. Conversely, patients with minor cardioembolism were the youngest and had the lowest central PP and AP. Patients with no cause had the lowest rates of hypertension, diabetes, and coronary artery disease but had the highest AP (all *p* values < 0.05) (Table 1).

**Table 1:** Demographic and clinical characteristics.

	Total (n = 746)	Arteriogenic embolism (n = 155)	Minor cardioembolism (n = 200)	Two more causes (n = 180)	No cause (n = 211)	*p-*value
Age, years	62.8 ± 13.1	66.8 ± 11.2	59.4 ± 13.9	65.9 ± 12.1	60.7 ± 13.3	< 0.001
Men	464 (62.2)	102 (65.8)	124 (62.0)	118 (65.6)	120 (56.9)	0.234
NIHSS score at admission	2.0 [1.0, 4.0]	2.0 [0.0, 4.0]	2.0 [1.0, 3.0]	2.0 [1.0, 4.0]	2.0 [0.0, 3.0]	0.705
Height, cm	163.6 ± 8.5	164.5 ± 7.6	164.4 ± 9.6	162.6 ± 8.3	163.0 ± 8.5	0.082
Body mass index, kg/m^2^	24.2 ± 3.3	24.7 ± 3.1	24.4 ± 3.6	23.9 ± 3.0	24.0 ± 3.4	0.108
Patent foramen ovale	329 (44.1)	0 (0.0)	175 (87.5)	154 (85.6)	0 (0.0)	< 0.001
MACE	100 (13.4)	30 (19.4)	19 (9.5)	24 (13.3)	27 (12.8)	0.060
Stroke recurrence	60 (8.0)	17 (11.0)	12 (6.0)	16 (8.9)	15 (7.1)	0.342
All-cause mortality	36 (4.8)	12 (7.7)	7 (3.5)	7 (3.9)	10 (4.7)	0.263
Infarct location						
Cerebral cortex	244 (32.7)	57 (36.8)	60 (30.0)	60 (33.3)	67 (31.8)	0.583
Cerebral subcortex	157 (21.0)	36 (23.2)	43 (21.5)	44 (24.4)	34 (16.1)	0.186
Insular	22 (2.9)	3 (1.9)	3 (1.5)	6 (3.3)	10 (4.7)	0.214
Corona radiata	1 (0.1)	0 (0.0)	1 (0.5)	0 (0.0)	0 (0.0)	0.435
Basal ganglia	182 (24.4)	37 (23.9)	56 (28.0)	42 (23.3)	47 (22.3)	0.561
Internal capsule	28 (3.8)	6 (3.9)	10 (5.0)	7 (3.9)	5 (2.4)	0.574
Thalamus	51 (6.8)	13 (8.4)	14 (7.0)	13 (7.2)	11 (5.2)	0.680
Midbrain	13 (1.7)	3 (1.9)	6 (3.0)	1 (0.6)	3 (1.4)	0.322
Pons	96 (12.9)	19 (12.3)	21 (10.5)	29 (3.9)	27 (12.8)	0.433
Medulla	33 (4.4)	7 (4.5)	7 (3.5)	5 (2.8)	14 (6.6)	0.261
Cerebellum	94 (12.6)	28 (18.1)	20 (10.0)	22 (12.2)	24 (11.4)	0.125
Acute treatments						
Thrombolysis	51 (6.8)	13 (8.4)	14 (7.0)	11 (6.1)	13 (6.2)	0.827
Thrombectomy	11 (1.5)	2 (1.3)	2 (1.0)	4 (2.2)	3 (1.4)	0.361
Risk factors						
Hypertension	541 (72.5)	126 (81.3)	144 (72.0)	144 (80.0)	127 (60.2)	< 0.001
Diabetes mellitus	203 (27.2)	56 (36.1)	53 (26.5)	53 (29.4)	41 (20.2)	0.004
Hypercholesterolemia	136 (18.2)	39 (25.2)	31 (15.5)	32 (17.8)	34 (16.1)	0.084
Current smoking	187 (25.1)	41 (26.5)	58 (29.0)	44 (24.4)	44 (20.9)	0.280
Coronary artery disease	264 (35.4)	57 (36.8)	71 (35.5)	86 (47.8)	50 (23.7)	< 0.001
Previous TIA/infarction	101 (13.5)	22 (14.2)	23 (11.5)	34 (18.9)	22 (10.4)	0.075
Laboratory findings						
Total cholesterol, mg/dL	180.1 ± 83.9	186.3 ± 102.7	175.6 ± 44.8	175.6 ± 45.1	183.2 ± 115.2	0.523
LDL-cholesterol, mg/dL	106.4 ± 38.0	107.6 ± 40.8	104.3 ± 37.7	108.3 ± 37.2	105.6 ± 36.3	0.714
HDL-cholesterol, mg/dL	43.5 ± 10.6	43.17 ± 10.0	44.8 ± 11.5	42.6 ± 10.7	43.4 ± 10.2	0.209
Triglyceride, mg/dL	126.0 ± 93.5	138.2 ± 127.9	126.1 ± 99.9	121.2 ± 67.2	120.0 ± 73.2	0.264
Secondary prevention						
Antiplatelet	743 (99.6)	155 (100.0)	198 (99.0)	180 (100.0)	210 (99.5)	0.564
Anticoagulant	9 (1.2)	0 (0.0)	4 (2.0)	0 (0.0)	5 (2.4)	0.036
Statin	579 (77.6)	126 (81.3)	153 (76.5)	141 (78.3)	159 (75.4)	0.568
Central BP measurements						
Admission to central BP, d	3.0 [2.0, 4.0]	3.0 [2.0, 4.0]	3.0 [2.0, 4.0]	3.0 [2.0, 4.0]	3.0 [2.0, 4.0]	0.369
Central SBP, mmHg	131.2 ± 21.1	131.6 ± 19.3	129.1 ± 21.2	131.5 ± 20.1	132.0 ± 23.2	0.509
Central DBP, mmHg	83.4 ± 13.6	82.5 ± 13.0	84.0 ± 13.7	82.7 ± 12.4	83.7 ± 15.0	0.635
Central PP, mmHg	47.7 ± 13.7	49.1 ± 13.3	45.0 ± 12.5	48.8 ± 13.4	48.2 ± 14.9	0.013
AP, mmHg	14.3 ± 8.1	15.0 ± 7.6	12.6 ± 7.4	14.7 ± 7.8	15.1 ± 9.0	0.008
AIx, %	24.1 ± 9.7	24.8 ± 8.5	22.7 ± 10.1	24.4 ± 9.6	24.6 ± 10.0	0.138

Data are expressed as mean ± standard deviation, median [interquartile range], or number (%). *AIx* augmentation index, *AP* augmentation pressure, *BP* blood pressure, *DBP* diastolic blood pressure, *HDL* high-density lipoprotein, *LDL* low-density lipoprotein; *MACE* major adverse cardiovascular event, *NIHSS* National Institutes of Health Stroke Scale, *PP* pulse pressure, *SBP* systolic blood pressure, *TIA* transient ischemic attack.

### Relationship between central BP and outcome

**Figure 2:**
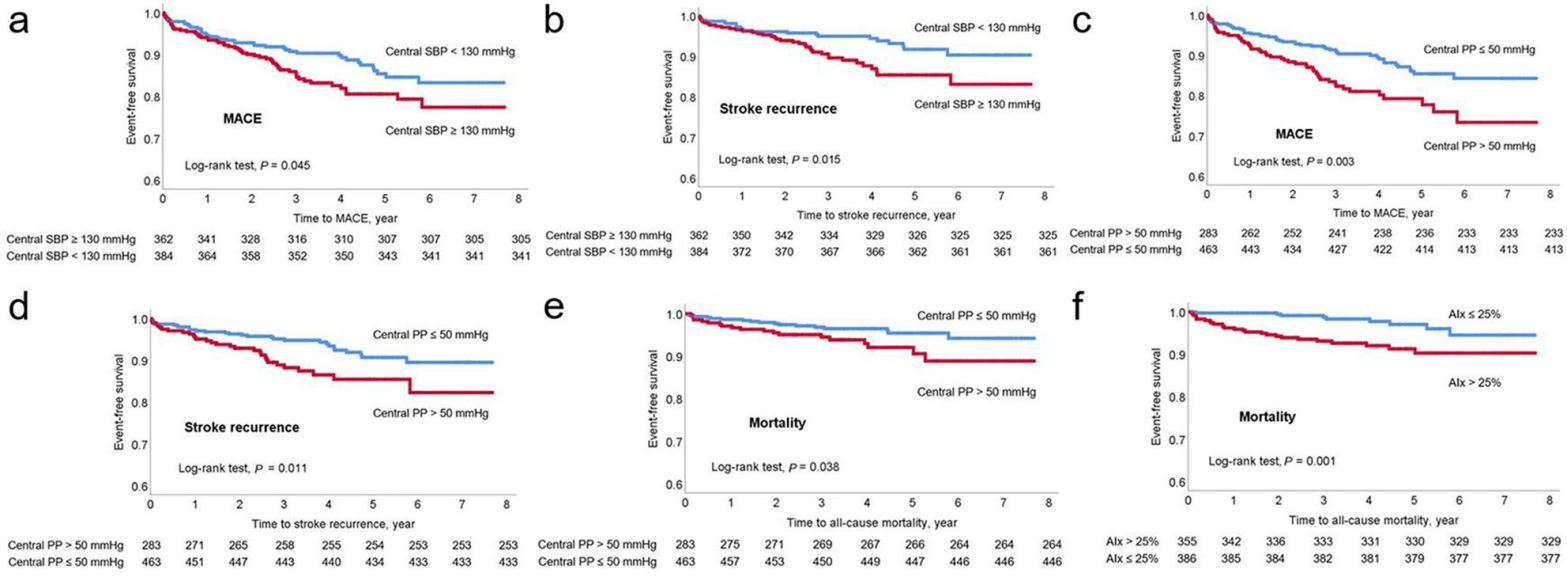
Kaplan–Meier survival plots for embolic stroke of undetermined source according to central blood pressure parameters. MACE (**a**) and stroke recurrence (**b**) according to central SBP. MACE (**c**), stroke recurrence (**d**), and all-cause mortality (**e**) according to central PP. All-cause mortality (**f**) according to AIx. *AIx* augmentation index, *MACE* major adverse cardiovascular event, *PP* pulse pressure, *SBP* systolic blood pressure.

The patients were followed up for a median of 43.4 months (interquartile range, 27.0–64.5 months). A total of 100 patients suffered MACE (13.4%), including 60 (8.0%) recurrent strokes, 10 (1.3%) acute coronary syndrome cases, 1 (0.1%) hospitalization for heart failure, and 29 (3.9%) deaths during the study period (Table 1). The Kaplan–Meier survival curves showed that central SBP ≥ 130 mmHg and PP > 50 mmHg were significantly associated with an increased risk of MACE and recurrent stroke (log-rank test; all *p* values < 0.05). All-cause mortality was significantly associated with central PP > 50 mmHg and AIx > 25% (log-rank test; all *p* values < 0.05) (Fig. 2).

Univariable Cox proportional hazards regression analysis showed that MACE was associated with old age, diabetes, no patent foramen ovale, central SBP, central SBP ≥ 130 mmHg, central PP, central PP > 50 mmHg, and AP. Recurrent stroke was significantly associated with old age, diabetes, no patent foramen ovale, central SBP, central SBP ≥ 130 mmHg, central PP, and central PP > 50 mmHg. All-cause mortality was significantly associated with old age, NIHSS score, diabetes, no patent foramen ovale, central PP, central PP > 50 mmHg, AP, AIx, and AIx > 25% (all *p* values < 0.05) (Supplementary Table 1).

Multivariable Cox proportional hazards regression analysis was performed to adjust for covariates (age, sex, NIHSS score, patent foramen ovale, hypertension, diabetes, coronary artery disease, anticoagulant, and ESUS subtype). Central SBP, PP, and PP > 50 mmHg were independently associated with an increased risk of MACE (central SBP: hazard ratio [HR] 1.014, 95% confidence interval [CI] 1.004‒1.024; central PP: HR 1.024, 95% CI 1.008‒1.040; central PP > 50 mmHg: HR 1.606, 95% CI 1.034‒2.496). In addition, central SBP, SBP ≥ 130 mmHg, DBP, and PP were independently associated with an increased risk of recurrent stroke (central SBP: HR 1.021, 95% CI 1.008‒1.034; central SBP ≥ 130 mmHg: HR 1.846, 95% CI 1.069‒3.188; central DBP: HR 1.022, 95% CI 1.002‒1.043; central PP: HR 1.029, 95% CI 1.009‒1.050). Meanwhile, all-cause mortality was associated with continuous and binary variables of AIx (AIx: HR 1.080, 95% CI 1.028‒1.134; AIx > 25%: HR 3.494, 95% CI 1.568‒7.782) (Table 2).

**Table 2:** Multivariable Cox regression analysis of long-term outcomes.

	MACE*		Stroke recurrence*		All-cause mortality*	
	HR (95% CI)	*p-*value	HR (95% CI)	*p-*value	HR (95% CI)	*p-*value
Central SBP, mmHg	1.014 (1.004‒1.024)	0.005	1.021 (1.008‒1.034)	0.001	1.000 (0.982‒1.017)	0.965
Central SBP ≥ 130 mmHg	1.407 (0.929‒2.131)	0.107	1.846 (1.069‒3.188)	0.028	1.047 (0.519‒2.109)	0.898
Central DBP, mmHg	1.012 (0.996‒1.028)	0.144	1.022 (1.002‒1.043)	0.028	0.980 (0.951‒1.009)	0.169
Central DBP ≥ 90 mmHg	1.501 (0.957‒2.354)	0.077	1.600 (0.899‒2.845)	0.110	0.987 (0.429‒2.268)	0.975
Central PP, mmHg	1.024 (1.008‒1.040)	0.003	1.029 (1.009‒1.050)	0.004	1.016 (0.991‒1.042)	0.221
Central PP > 50 mmHg	1.606 (1.034‒2.496)	0.035	1.657 (0.937‒2.931)	0.082	1.468 (0.693‒3.112)	0.317
AP, mmHg	1.022 (0.996‒1.049)	0.101	1.018 (0.983‒1.053)	0.319	1.032 (0.989‒1.076)	0.145
AP > 13 mmHg	1.219 (0.801‒1.854)	0.356	1.490 (0.862‒2.577)	0.153	1.259 (0.620‒2.555)	0.524
AIx, %	1.017 (0.992‒1.043)	0.174	0.990 (0.961‒1.020)	0.512	1.080 (1.028‒1.134)	0.002
AIx > 25%	1.497 (0.983‒2.279)	0.060	1.058 (0.619‒1.806)	0.837	3.494 (1.568‒7.782)	0.002

*Adjusted for age, sex, NIHSS score at admission, patent foramen ovale, hypertension, diabetes mellitus, coronary artery disease, anticoagulant, and ESUS subtype. *AIx* augmentation index, *AP* augmentation pressure, *CI* confidence interval, *DBP* diastolic blood pressure, *ESUS* embolic stroke of undetermined source, *HR* hazard ratio, *MACE* major adverse cardiovascular event, *NIHSS* National Institutes of Health Stroke Scale, *PP* pulse pressure, *SBP* systolic blood pressure.

**Figure 3:**
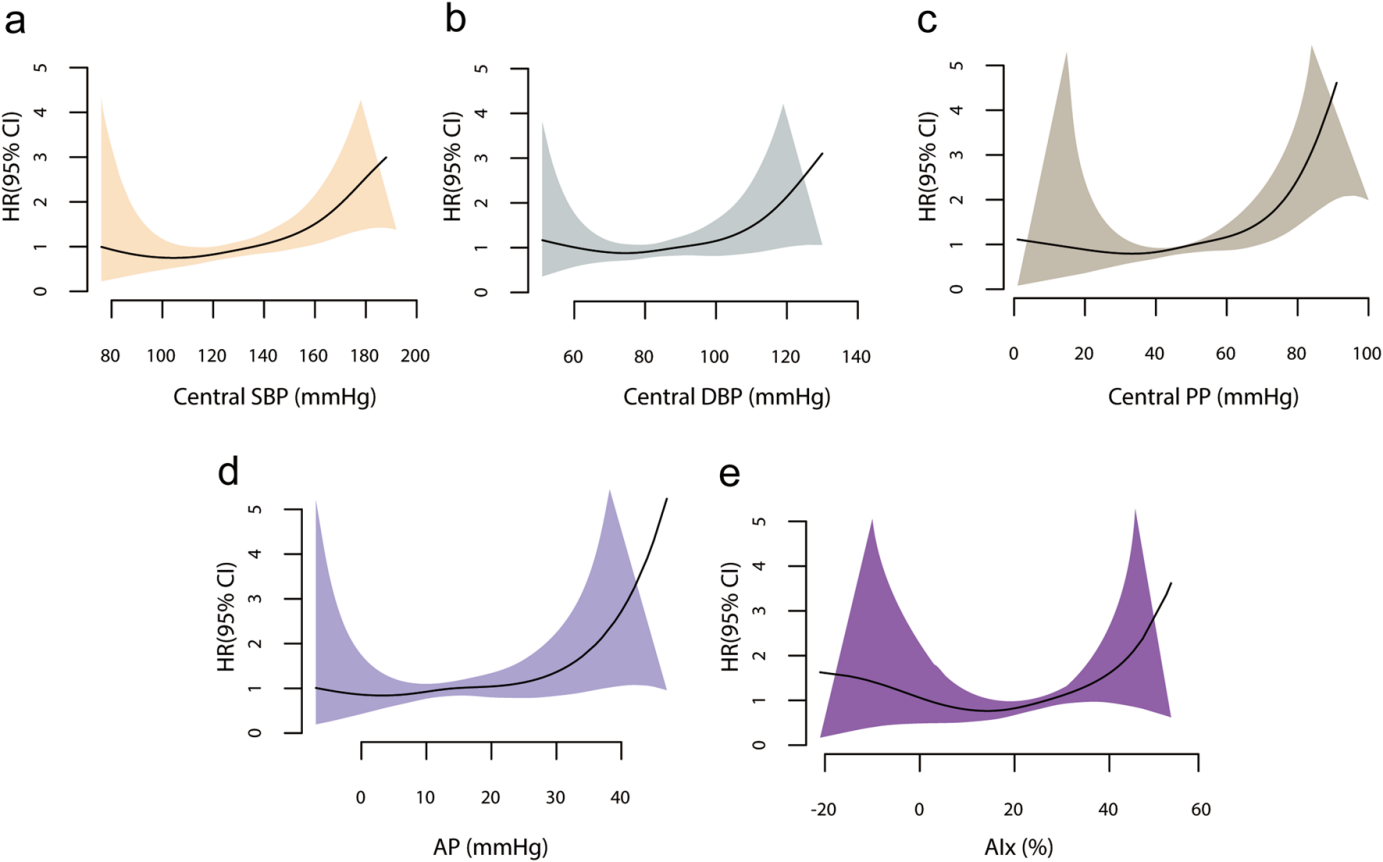
Restricted cubic spline curves of the risk for the primary outcome. HRs (line) and 95% CIs (shading) of MACE associated with central SBP (**a**), central DBP (**b**), central PP (**c**), AP (**d**), and AIx (**e**) were calculated after adjusting for age, sex, NIHSS score at admission, patent foramen ovale, hypertension, diabetes mellitus, coronary artery disease, anticoagulant, and ESUS subtype. *AIx* augmentation index, *AP* augmentation pressure, *CI* confidence interval, *DBP* diastolic blood pressure, *ESUS* embolic stroke of undetermined source, *HR* hazard ratio, *MACE* major adverse cardiovascular event, *NIHSS* National Institutes of Health Stroke Scale, *PP* pulse pressure, *SBP* systolic blood pressure.

The impact of central BP parameters on the risk of the primary outcome is visualized in Fig. 3. Restricted cubic spline analysis for the HR of central BP showed that the adjusted risk for MACE increased with higher central BP parameters above the cutoff value.

### Prognostic impact of central BP by ESUS subtype

We further conducted multivariable Cox regression analysis based on the subtype of ESUS. Among the subtypes, no cause ESUS showed a strong association between central BP parameters and long-term outcomes. The cutoff values for central SBP, central PP, AP, and AIx showed an independent association with MACE (central SBP ≥ 130 mmHg: HR 2.690, 95% CI 1.100‒6.578; central PP > 50 mmHg: HR 6.943, 95% CI 2.560‒18.834; AP > 13 mmHg: HR 4.929, 95% CI 1.869‒13.001; AIx > 25%: HR 3.952, 95% CI 1.638‒9.534). The cutoff values for central SBP, central PP, and AP showed an independent association with recurrent stroke (central SBP ≥ 130 mmHg: HR 5.126, 95% CI 1.309‒20.077; central PP > 50 mmHg: HR 9.669, 95% CI 2.262‒41.319; AP > 13 mmHg: HR 7.797, 95% CI 1.826‒33.296). Furthermore, the cutoff values of AP and AIx showed an independent association with all-cause mortality (AP > 13 mmHg: HR 6.539, 95% CI 1.151‒37.144; AIx > 25%: HR 9.744, 95% CI 1.561‒60.813) (Table 3).

**Table 3:** Multivariable Cox regression analysis of long-term outcomes according to the characteristics of ESUS subtype.

	MACE*		Stroke recurrence*		All-cause mortality*	
	HR (95% CI)	*p-*value	HR (95% CI)	*p-*value	HR (95% CI)	*p-*value
Arteriogenic embolism (n = 155)						
Central SBP ≥ 130 mmHg	1.131 (0.519‒2.464)	0.757	0.920 (0.328‒2.582)	0.874	1.107 (0.324‒3.782)	0.872
Central DBP ≥ 90 mmHg	1.739 (0.757‒3.991)	0.192	1.307 (0.428‒3.987)	0.638	1.928 (0.482‒7.719)	0.353
Central PP > 50 mmHg	0.935 (0.412‒2.118)	0.871	0.894 (0.307‒2.605)	0.838	0.677 (0.169‒2.714)	0.582
AP > 13 mmHg	0.502 (0.232‒1.088)	0.081	0.738 (0.273‒1.996)	0.550	0.239 (0.057‒0.992)	0.049
AIx > 25%	1.096 (0.489‒2.459)	0.823	0.963 (0.345‒2.688)	0.943	1.170 (0.289‒4.738)	0.826
Minor cardioembolism (n = 200)						
Central SBP ≥ 130 mmHg	1.258 (0.472‒3.350)	0.646	3.324 (0.832‒13.284)	0.089	0.982 (0.185‒5.212)	0.983
Central DBP ≥ 90 mmHg	1.280 (0.461‒3.549)	0.636	1.478 (0.403‒5.422)	0.556	0.487 (0.051‒4.617)	0.531
Central PP > 50 mmHg	0.587 (0.185‒1.867)	0.367	0.866 (0.207‒3.634)	0.845	1.149 (0.188‒7.031)	0.880
AP > 13 mmHg	1.525 (0.550‒4.225)	0.417	1.948 (0.530‒7.158)	0.315	3.950 (0.481‒32.448)	0.201
AIx > 25%	1.419 (0.509‒3.956)	0.503	1.797 (0.479‒6.737)	0.385	7.367 (0.612‒88.706)	0.116
Two more causes (n = 180)						
Central SBP ≥ 130 mmHg	1.077 (0.475‒2.439)	0.859	1.448 (0.524‒3.999)	0.475	1.520 (0.324‒7.122)	0.595
Central DBP ≥ 90 mmHg	2.264 (0.877‒5.845)	0.091	2.292 (0.745‒7.056)	0.148	1.045 (0.117‒9.306)	0.969
Central PP > 50 mmHg	1.291 (0.544‒3.066)	0.562	1.268 (0.439‒3.665)	0.661	2.825 (0.514‒15.517)	0.232
AP > 13 mmHg	0.824 (0.360‒1.888)	0.648	0.862 (0.310‒2.393)	0.775	2.163 (0.406‒11.538)	0.366
AIx > 25%	0.985 (0.428‒2.375)	0.985	0.441 (0.147‒1.322)	0.144	17.928 (1.760‒182.668)	0.015
No cause (n = 211)						
Central SBP ≥ 130 mmHg	2.690 (1.100‒6.578)	0.030	5.126 (1.309‒20.077)	0.019	1.274 (0.299‒5.429)	0.743
Central DBP ≥ 90 mmHg	0.909 (0.336‒2.460)	0.851	1.149 (0.309‒4.277)	0.836	0.794 (0.137‒4.616)	0.798
Central PP > 50 mmHg	6.943 (2.560‒18.834)	< 0.001	9.669 (2.262‒41.319)	0.002	4.557 (0.859‒24.168)	0.075
AP > 13 mmHg	4.929 (1.869‒13.001)	0.001	7.797 (1.826‒33.296)	0.006	6.539 (1.151‒37.144)	0.034
AIx > 25%	3.952 (1.638‒9.534)	0.002	2.614 (0.852‒8.023)	0.093	9.744 (1.561‒60.813)	0.015

*Adjusted for age, sex, NIHSS score at admission, patent foramen ovale, hypertension, diabetes mellitus, coronary artery disease, and anticoagulant. *AIx* augmentation index, *AP* augmentation pressure, *CI* confidence interval, *DBP* diastolic blood pressure, *HR* hazard ratio, *MACE* major adverse cardiovascular event, *NIHSS* National Institutes of Health Stroke Scale, *PP* pulse pressure, *SBP* systolic blood pressure.

## Discussion

We demonstrated that central BP parameters independently predicted poor long-term outcomes in patients with ESUS. Central SBP and PP were particularly associated with MACE and recurrent stroke, whereas AIx was linked to all-cause mortality. Interestingly, among the ESUS subtypes, patients with no cause ESUS showed a strong association between central BP and outcomes. Therefore, our study suggests that central BP is a useful prognostic marker for patients with ESUS, especially those with the no cause subtype.

Central BP is easily and noninvasively derived from radial artery waveform. Applanation tonometry is commonly used to detect radial artery waveform because of its simplicity and good tolerability. This tonometry utilizes a transfer function to calculate the aortic pressure waveform from the radial artery. The aortic pressure waveform can yield central BP parameters that reflect both arterial stiffness and wave reflection^20^.

Blood vessels lose flexibility and become stiffer with age, which impedes heart function. This is known as arterial stiffness. Arterial stiffness results from aging and arteriosclerosis. Inflammation also plays a major role in arteriosclerosis development and is consequently a major contributor to large artery stiffening^21^. Arterial stiffness is a well-established intermediate endpoint for cardiovascular outcome because it increases the risk of cardiovascular events, such as myocardial infarction, hypertension, heart failure, and stroke^22^. Our previous study also showed that increased stiffness predicted poor long-term prognosis in patients with cryptogenic stroke^23^. In stiff arteries, the reflected wave arrives back at the central arteries earlier and amplifies the forward wave, thereby augmenting the systolic pressure. A premature return of reflected waves in late systole increases the left ventricular load and myocardial oxygen demand, resulting in left ventricular hypertrophy^24^. Thus, both wave reflection and aortic stiffness are closely related to unfavorable outcomes^25–27^.

Previous studies have proven that central SBP and PP are significantly associated with an increased risk of subclinical cerebrovascular disease and future stroke^10,28^. An increased AIx was also associated with in-hospital outcomes, such as longer length of stay and lower Barthel index score in patients with ischemic stroke^29^. Similarly, prospective studies revealed that an increased AIx was an independent predictor of poor functional outcome after acute ischemic stroke^12,13^. In line with these reports, we found that central BP parameters were independently associated with poor long-term outcomes in patients with ESUS.

Poor long-term outcome may result from worsened aortic stiffness and wave reflection, which can be measured by central BP. We hypothesize possible mechanisms like below. First, increased central BP influences arterial remodeling in both the extracranial and intracranial arteries, which may result in increased carotid wall thickness^30^, the development of stenosis and plaques^31^, and the likelihood of plaque rupture^32^. Second, blood flow impedance in the cerebral artery is lower than that in other systemic arteries. Aortic stiffness can facilitate the transmission of pulsatile pressure into the cerebral microcirculation^33^. Furthermore, torrential flow and high-pressure fluctuations in the carotid and vertebral arteries can induce cerebrovascular damage^11^. Third, aortic stiffness and wave reflection are associated with poor left ventricular conditions, which may increase the risk of poor outcomes^34^. Fourth, aortic stiffness is linked to atherosclerosis because both share several risk factors, including hypertension^35^. The current study showed that hypertension was common in 72.5% of patients. Thus, atherosclerosis may contribute to a poor prognosis in ESUS in patients with high central BP.

A previous meta-analysis showed that central SBP and PP and AIx were significantly predictive of MACE, whereas all-cause mortality was associated only with AIx^36^. Similarly, we found that central SBP and PP were associated with MACE and stroke recurrence, whereas AIx was associated with all-cause mortality. The underlying cause for this is unclear, but it may arise from the fact that AIx values were negative in 18 (2.4%) patients in our cohort. Wave intensity analysis indicates that negative AIx is principally due to a forward travelling (re-reflected) decompression wave in mid-systole and should not be used as an estimate of wave reflection magnitude^37^. This loophole may attenuate the prognostic effect of AIx for MACE, including stroke recurrence. Additionally, ESUS patients with high central SBP or PP had higher levels of vascular risk factors including total cholesterol, triglyceride, and body mass index than those with high AIx (Supplementary Table 2). These factors may cause differences in the prognostic impact among central BP parameters. However, given the conflicting and limited data regarding the association between central BP and outcomes in ESUS patients, more studies are needed to define the predictive value of central BP in the population with ESUS.

Central BP parameters showed strong prognostic values for poor outcomes in no cause ESUS. This is an unexpected finding because no cause ESUS usually involves a hidden embolic source, such as atrial fibrillation. Conversely, central BP is a parameter of arterial stiffness and wave reflection. Our results suggest several potential reasons for this. First, patients with no cause ESUS had the highest AP among the subtypes. Previous studies have reported that increased arterial stiffness and wave reflection are related to newly developed atrial fibrillation^38^. Given the high AP, hidden atrial fibrillation may be involved in poor prognosis in no cause ESUS. In addition, AP more sensitively reflects ventricular ejection functions than other central BP parameters^39^, and thus high AP may precipitate the risk of recurrent stroke due to left ventricular dysfunction^40^. Second, patients with other ESUS subtypes were older and had higher rates of hypertension, diabetes, and coronary artery disease than those with no cause ESUS. These risk factors are well-known prognostic markers after stroke. Although we adjusted for these factors in the multivariable analysis, risk factors may increase the likelihood of poor outcomes in other ESUS subtypes than no cause ESUS and mitigate the effect size of central BP parameters on clinical outcomes^41^.

Our study has noteworthy strengths. We enrolled patients with comprehensive work-ups, including TEE and prolonged electrocardiography monitoring. All patients underwent TEE and transthoracic echocardiography was performed in 93.2% of patients. Prolonged heart rhythm evaluation was also performed in 93.6% of patients (Supplementary Table 3). ESUS working group investigators recommended a comprehensive stroke work-up, but they did not consider TEE and long-term electrocardiography monitoring as mandatory investigations^42^. TEE examination can uncover abnormal findings in more than half of cases, and such abnormalities significantly impact on the prognosis of ESUS patients^43^. Another strength is that we measured central BP using a device readily available in clinical practice. Measuring central BP is simple, non-invasive, and chief. Based on our finding on the prognostic value of central BP in ESUS, the central BP measurement may be recommended for identifying high-risk patients without an obvious etiology.

This study has several limitations. First, although we included consecutive patients with ESUS, the mandatory inclusion criteria may have excluded some patients with ESUS who did not undergo central BP measurement and TEE. Second, central BP may be affected by the type and dosage of antihypertensive medication^26^. We did not analyze these factors because too many types of antihypertensive drugs were used. Third, we only assessed patients of a single ethnicity and from a single center. Further prospective validation studies involving other ethnicities and larger cohorts are required. Despite these limitations, our study firstly reported the association between central BP and long-term prognosis of ESUS patients.

## Conclusions

We found that central BP parameters in patients with ESUS were independently associated with unfavorable outcomes, including MACE, stroke recurrence, and mortality. The prognostic impact of these parameters was most evident in patients with no cause ESUS. Therefore, we propose that central BP measurement may be used as an important diagnostic and prognostic factor for patients with ESUS, especially those with no apparent embolic cause.

## Supplementary Information

Supplementary Tables.

## Data Availability

The study data are available from the corresponding author upon reasonable request and with the permission of all contributing authors.

## Abbreviations

AIx: Augmentation index
AP: Augmentation pressure
DBP: Diastolic blood pressure
ESUS: Embolic stroke of undetermined source
PP: Pulse pressure
SBP: Systolic blood pressure
TEE: Transesophageal echocardiography
