# Supplementary material for: Prognostic value of central blood pressure on the outcomes of embolic stroke of undetermined source
Source: Sci Rep. 2023 Jun 12;13:9550. doi: 10.1038/s41598-023-36151-y (PMC10261100; doi:10.1038/s41598-023-36151-y)
Supplement: Supplementary file 1 — Supplementary Tables. [file 41598_2023_36151_MOESM1_ESM.docx]

**Supplementary Table 1. Univariable Cox regression analysis of long-term outcomes.**

|  | MACE |  | Stroke recurrence |  | All-cause mortality |  |
| --- | --- | --- | --- | --- | --- | --- |
|  | HR (95% CI) | *p-*value | HR (95% CI) | *p-*value | HR (95% CI) | *p-*value |
| Age, y | 1.025 (1.008‒1.042) | 0.003 | 1.020 (1.007‒1.051) | 0.010 | 1.043 (1.013‒1.073) | 0.005 |
| Men | 1.467 (0.956‒2.250) | 0.079 | 1.560 (0.890‒2.736) | 0.121 | 1.574 (0.759‒3.264) | 0.223 |
| NIHSS score at admission | 1.048 (0.996‒1.102) | 0.069 | 1.025 (0.953‒1.102) | 0.506 | 1.116 (1.048‒1.188) | 0.001 |
| Patent foramen ovale | 0.484 (0.316‒0.740) | 0.001 | 0.581 (0.341‒0.989) | 0.046 | 0.432 (0.208‒0.897) | 0.024 |
| Hypertension | 1.402 (0.879‒2.234) | 0.156 | 1.266 (0.705‒2.273) | 0.429 | 1.727 (0.756‒3.945) | 0.195 |
| Diabetes mellitus | 1.741 (1.160‒2.612) | 0.007 | 2.096 (1.255‒3.503) | 0.005 | 2.042 (1.053‒3.961) | 0.035 |
| Coronary artery disease | 1.307 (0.877‒1.947) | 0.189 | 1.425 (0.854‒2.375) | 0.175 | 0.929 (0.464‒1.860) | 0.836 |
| Anticoagulant | 0.049 (0.000‒172.751) | 0.469 | 0.049 (0.000‒1988.161) | 0.577 | 0.049 (0.000‒51482.089) | 0.670 |
| ESUS subtypes |  |  |  |  |  |  |
| No cause | Ref |  | Ref |  | Ref |  |
| Arteriogenic embolism | 1.603 (0.953‒2.696) | 0.075 | 1.673 (0.835‒3.352) | 0.146 | 1.706 (0.737‒3.948) | 0.213 |
| Minor cardioembolism | 0.673 (0.374‒1.212) | 0.187 | 0.760 (0.356‒1.625) | 0.479 | 0.689 (0.262‒1.811) | 0.450 |
| Two more causes | 0.956 (0.551‒1.657) | 0.872 | 1.148 (0.567‒2.323) | 0.701 | 0.762 (0.290‒2.004) | 0.582 |
| Central SBP, mmHg | 1.012 (1.003‒1.022) | 0.007 | 1.017 (1.006‒1.029) | 0.003 | 1.003 (0.987‒1.018) | 0.743 |
| Central SBP ≥ 130 mmHg | 1.496 (1.007‒2.224) | 0.046 | 1.891 (1.122‒3.185) | 0.017 | 1.254 (0.651‒2.414) | 0.499 |
| Central DBP, mmHg | 1.004 (0.989‒1.018) | 0.609 | 1.009 (0.992‒1.027) | 0.282 | 0.974 (0.948‒1.001) | 0.057 |
| Central DBP ≥ 90 mmHg | 1.239 (0.811‒1.894) | 0.321 | 1.270 (0.737‒2.188) | 0.390 | 0.776 (0.354‒1.703) | 0.527 |
| Central PP, mmHg | 1.025 (1.012‒1.039) | <0.001 | 1.030 (1.013‒1.047) | 0.001 | 1.026 (1.004‒1.048) | 0.018 |
| Central PP > 50 mmHg | 1.812 (1.224‒2.684) | 0.003 | 1.904 (1.146‒3.163) | 0.013 | 1.972 (1.024‒3.798) | 0.042 |
| AP, mmHg | 1.026 (1.002‒1.050) | 0.032 | 1.023 (0.992‒1.054) | 0.146 | 1.042 (1.005‒1.081) | 0.026 |
| AP > 13 mmHg | 1.355 (0.913‒2.012) | 0.132 | 1.654 (0.986‒2.775) | 0.057 | 1.489 (0.767‒2.890) | 0.239 |
| AIx, % | 1.016 (0.994‒1.038) | 0.154 | 0.996 (0.970‒1.022) | 0.755 | 1.062 (1.020‒1.106) | 0.003 |
| AIx > 25% | 1.460 (0.981‒2.170) | 0.062 | 1.099 (0.662‒1.824) | 0.715 | 3.330 (1.560‒7.108) | 0.002 |

*AIx* augmentation index, *AP* augmentation pressure, *CI* confidence interval, *DBP* diastolic blood pressure, *ESUS* embolic stroke of undetermined source, *HR* hazard ratio, *MACE* major adverse cardiovascular event, *NIHSS* National Institutes of Health Stroke Scale, *PP* pulse pressure, *SBP* systolic blood pressure.

**Supplementary Table 2. Patient demographic and clinical characteristics.**

|  | Central SBP ≥ 130 mmHg  (n = 362) | Central SBP < 130 mmHg  (n = 384) | *p-*value | Central PP > 50 mmHg  (n = 283) | Central PP ≤ 50 mmHg  (n = 463) | *p-*value | AIx > 25%  (n = 355) | AIx ≤ 25%  (n = 386) | *p-*value |
| --- | --- | --- | --- | --- | --- | --- | --- | --- | --- |
| Age, year | 64.2±12.7 | 61.6±13.4 | 0.005 | 67.9±11.3 | 59.8±13.2 | <0.001 | 65.4±12.0 | 60.4±13.6 | <0.001 |
| Men | 216 (59.7) | 248 (64.6) | 0.166 | 147 (51.9) | 317 (68.5) | <0.001 | 170 (47.9) | 292 (75.6) | <0.001 |
| NIHSS score at admission | 2.0 [1.0, 3.0] | 2.0 [0.0, 4.0] | 0.329 | 2.0 [1.0, 3.0] | 2.0 [1.0, 4.0] | 0.672 | 2.0 [1.0, 4.0] | 2.0 [0.0, 3.0] | 0.023 |
| Height, cm | 163.5±8.5 | 163.6±8.7 | 0.887 | 161.8±8.3 | 164.6±8.7 | <0.001 | 160.9±8.1 | 166.0±8.3 | <0.001 |
| Body mass index, kg/m^2^ | 24.7±3.6 | 23.8±2.9 | <0.001 | 24.4±3.4 | 24.2±3.3 | 0.377 | 24.0±3.3 | 24.5±3.2 | 0.059 |
| **Acute treatments** |  |  |  |  |  |  |  |  |  |
| Thrombolysis | 25 (6.9) | 26 (6.8) | 0.942 | 20 (7.1) | 31 (6.7) | 0.845 | 29 (8.2) | 22 (5.7) | 0.185 |
| Thrombectomy | 3 (0.8) | 8 (2.1) | 0.155 | 2 (0.7) | 9 (1.9) | 0.222 | 3 (0.8) | 8 (2.1) | 0.141 |
| **Risk factors** |  |  |  |  |  |  |  |  |  |
| Hypertension | 299 (82.6) | 242 (63.0) | <0.001 | 242 (85.8) | 299 (64.6) | <0.001 | 272 (76.6) | 265 (87.7) | 0.015 |
| Diabetes mellitus | 112 (30.9) | 91 (23.7) | 0.026 | 97 (34.3) | 106 (22.9) | 0.001 | 114 (32.1) | 88 (22.8) | 0.004 |
| Hypercholesterolemia | 73 (20.2) | 63 (16.4) | 0.184 | 56 (19.8) | 80 (17.3) | 0.389 | 71 (20.0) | 65 (16.8) | 0.267 |
| Current smoking | 93 (25.7) | 94 (24.5) | 0.703 | 58 (20.5) | 129 (27.9) | 0.024 | 85 (23.9) | 102 (26.4) | 0.437 |
| Coronary artery disease | 124 (34.3) | 140 (36.5) | 0.529 | 100 (35.3) | 164 (35.4) | 0.981 | 125 (35.2) | 137 (35.5) | 0.939 |
| Previous TIA/infarction | 49 (13.5) | 52 (13.5) | 0.998 | 40 (14.1) | 61 (13.2) | 0.710 | 44 (12.4) | 56 (14.5) | 0.400 |
| **Laboratory findings** |  |  |  |  |  |  |  |  |  |
| Total cholesterol, mg/dL | 186.9±110.8 | 173.3±43.5 | 0.026 | 178.5±103.5 | 180.8±68.5 | 0.712 | 183.0±94.4 | 177.4±72.6 | 0.367 |
| LDL-cholesterol, mg/dL | 108.3±37.7 | 104.4±37.9 | 0.157 | 104.4±38.1 | 107.5±37.7 | 0.292 | 108.1±37.5 | 104.8±38.3 | 0.236 |
| HDL-cholesterol, mg/dL | 43.3±10.4 | 43.8±10.9 | 0.485 | 43.1±10.3 | 43.8±10.9 | 0.325 | 44.0±10.5 | 43.1±10.8 | 0.237 |
| Triglyceride, mg/dL | 136.2±115.5 | 115.8±64.2 | 0.003 | 126.9±109.5 | 125.0±81.8 | 0.788 | 127.3±100.2 | 124.3±86.7 | 0.654 |
| **Secondary prevention** |  |  |  |  |  |  |  |  |  |
| Antiplatelet | 361 (99.7) | 382 (99.5) | 1.000 | 283 (100.0) | 460 (99.4) | 0.293 | 355 (100) | 383 (99.2) | 0.250 |
| Anticoagulant | 6 (1.7) | 3 (0.8) | 0.328 | 5 (1.8) | 4 (0.9) | 0.311 | 5 (1.4) | 4 (1.0) | 0.744 |
| Statin | 284 (78.5) | 295 (76.8) | 0.593 | 213 (75.3) | 366 (79.0) | 0.229 | 275 (77.5) | 300 (77.7) | 0.934 |

Significant intergroup differences were analyzed by independent two-sample t-test or the Mann–Whitney U test for continuous variables and chi-square test or Fisher’s exact test for categorical variables. Data are expressed as mean ± standard deviation, median [interquartile range], or number (%). *AIx* augmentation index, *HDL* high-density lipoprotein, *LDL* low-density lipoprotein, *NIHSS* National Institutes of Health Stroke Scale, *PP* pulse pressure, *SBP* systolic blood pressure, *TIA* transient ischemic attack.

**Supplementary Table 3. Etiologic evaluations according to ESUS subtypes.**

|  | Total  (n = 746) | Arteriogenic embolism  (n = 155) | Minor cardioembolism  (n = 200) | Two or more causes  (n = 180) | No cause  (n = 211) | *p-*value |
| --- | --- | --- | --- | --- | --- | --- |
| Angiographic evaluations | 718 (96.2) | 149 (96.1) | 197 (98.5) | 178 (98.9) | 194 (91.9) | 0.001 |
| CTA | 544 (72.9) | 112 (72.3) | 151 (75.5) | 138 (76.7) | 143 (67.8) | 0.185 |
| MRA | 668 (89.5) | 133 (85.8) | 190 (95.0) | 167 (92.8) | 178 (84.4) | 0.001 |
| DSA | 93 (12.5) | 22 (14.2) | 18 (9.0) | 30 (16.7) | 23 (10.9) | 0.111 |
| Neurosonographic evaluations | 716 (96.0) | 148 (95.5) | 195 (97.5) | 178 (98.9) | 195 (92.4) | 0.007 |
| Carotid Doppler | 663 (88.9) | 140 (90.3) | 176 (88.0) | 164 (91.1) | 183 (86.7) | 0.498 |
| TCD | 669 (89.7) | 135 (87.1) | 186 (93.0) | 164 (91.1) | 184 (87.2) | 0.151 |
| Echocardiography |  |  |  |  |  |  |
| TEE | 746 (100.0) | 155 (100.0) | 200 (100.0) | 180 (100.0) | 211 (100.0) | NA |
| TTE | 695 (93.2) | 143 (92.3) | 196 (98.0) | 174 (96.7) | 182 (83.6) | <0.001 |
| Heart rhythm evaluations | 698 (93.6) | 144 (92.9) | 183 (91.5) | 168 (93.3) | 203 (96.2) | 0.262 |
| Continuous ECG monitoring | 635 (85.1) | 132 (85.2) | 164 (82.0) | 155 (86.1) | 184 (87.2) | 0.494 |
| Holter | 378 (50.7) | 80 (51.6) | 98 (49.0) | 72 (40.0) | 128 (60.7) | 0.001 |
| Implantable loop recorder | 8 (1.1) | 1 (0.6) | 1 (0.5) | 0 (0.0) | 6 (2.8) | 0.028 |
| Heart CT | 493 (66.1) | 94 (60.6) | 157 (78.5) | 118 (65.6) | 124 (58.8) | <0.001 |

Data are expressed as number (%). *CT* computed tomography, *CTA* computed tomography angiography, *DSA* digital subtraction angiography, *ECG* electrocardiogram, *ESUS* embolic stroke of undetermined source, *MRA* magnetic resonance angiography, *NA* not analyzed, *TCD* transcranial Doppler, *TEE* transesophageal echocardiography, *TTE* transthoracic echocardiography.
